# Synthesis of Uniform Size Rutile TiO_2_ Microrods by Simple Molten-Salt Method and Its Photoluminescence Activity

**DOI:** 10.3390/nano12152626

**Published:** 2022-07-29

**Authors:** Hieu Minh Ngo, Amol Uttam Pawar, Jun Tang, Zhongbiao Zhuo, Don Keun Lee, Kang Min Ok, Young Soo Kang

**Affiliations:** 1 Department of Chemistry, Sogang University, Seoul 04107, Korea; hieungo@sogang.ac.kr (H.M.N.); kmok@sogang.ac.kr (K.M.O.); 2 Environmental and Climate Technology, Korea Institute of Energy Technology, Naju-si 58219, Korea; amolphysics@kentech.ac.kr (A.U.P.); leedk3@kentech.ac.kr (D.K.L.); 3 Zhejiang Coloray Technology Development Co., Ltd., No. 151, Huishan Road, Deqing County, Huzhou 313200, China; tangjun@coloray.com.cn (J.T.); zhongbiao@coloray.com.cn (Z.Z.)

**Keywords:** molten-salt method, TiO_2_ microrod, anatase, rutile, photoluminescence

## Abstract

Uniform-size rutile TiO_2_ microrods were synthesized by simple molten-salt method with sodium chloride as reacting medium and different kinds of sodium phosphate salts as growth control additives to control the one-dimensional (1-D) crystal growth of particles. The effect of rutile and anatase ratios as a precursor was monitored for rod growth formation. Apart from uniform rod growth study, optical properties of rutile microrods were observed by UV−visible and photoluminescence (PL) spectroscopy. TiO_2_ materials with anatase and rutile phase show PL emission due to self-trapped exciton. It has been observed that synthesized rutile TiO_2_ rods show various PL emission peaks in the range of 400 to 900 nm for 355 nm excitation wavelengths. All PL emission appeared due to the oxygen vacancy present inside rutile TiO_2_ rods. The observed PL near the IR range (785 and 825 nm) was due to the formation of a self-trapped hole near to the surface of (110) which is the preferred orientation plane of synthesized rutile TiO_2_ microrods.

## 1. Introduction

The uniform shape and size of nano/microparticles with selective crystal facets are always challenging for synthesis, and those uniform particles are very useful for many applications. In chromatography applications, uniform particle size shows good resolution, better packing properties, improved kinetics, and it maintains consistent performance. It also can allow even and homogeneous coating on the solid surfaces [1]. In the field of electrochemistry, Fuller et al. suggested the improved stability of Pt in the membrane due to uniform particle size distribution [2]. Similarly, in paint industries many properties also depend upon the particle size distribution, such as transparency, film color appearance, paint viscosity, color stability, and weather resistance. In short, it is understood that more uniform particle size shows better performance in many fields of applications in modern life.

TiO_2_ is one of the versatile materials that can be used in various fields due to its unique optical and electrical properties. Hence, synthesis of uniform size and shape with selective crystal facets of TiO_2_ particles is key in the current research field. There are different chemical methods used for the synthesis of uniform micro- and nanoparticles, for example, the micro-emulsion method [3], co-precipitation method [4], sol-gel method [5], and solvothermal/hydrothermal method with different surfactant and capping agents [6]. Kang et al. reported a truncated and rice-like one-axis {001}-oriented crystalline anatase TiO_2_ by simple hydrothermal synthesis. It was presumed that {001} facets were highly reactive and showed better catalytic performance compared to other crystal facets [7]. Hence, shape variation can vary the active surface area, which further causes change in catalytic performance. However, not only the shape but size of nano-microparticles also shows variation in its catalytic performance. Hao et al. carried out a comparative photodegradation study of rhodamine B with TiO_2_ nanoparticles for different particle sizes of 8, 16, and 150 nm. It was observed that a smaller particle size gave better performance in a photodegradation study [8]. Chen et al. reported uniform coating of TiO_2_ nanoparticles with sizes of 5 to 10 nm on natural cellulose by the solvothermal method, which showed an enhanced photocatalytic performance in the field of dye degradation study [9]. However, it is not only smaller size particles that always produce better performance; many times it is completely dependent on the application. For example, in the paint industry, pigment properties mostly depend upon the light scattering which further depends upon wavelength and particle size. For efficient light scattering, the diameter of the particles should be slightly smaller than the half wavelength of incident light. For example, for visible light range from 380 to 750 nm, the particle size should be slightly less than or half of that range, which is about 200 to 300 nm and much higher than earlier examples [10]. Moreover, TiO_2_ is an outstanding material with a high refractive index, which is the main reason for light scattering and one of the important parameters for white pigments. Among different phases of TiO_2_, rutile TiO_2_ shows a 10% higher refractive index than anatase TiO_2_ [11]. Furthermore, by considering various morphologies, one-dimensional (1-D) structures such as rods, belts, wires, and fibers show excellent mechanical and electrical properties with high surface to volume ratio [12]. Finally, it was decided to synthesize rutile TiO_2_ rods with a diameter approximately 300 nm with longer length (in µm) and uniform size, which will suit applications in the paint industry.

There are several reports published on the synthesis of 1-D TiO_2_ with different synthesis techniques and for various applications [13,14,15]. The molten-salt synthesis technique is one of the promising techniques and highly accepted for rutile TiO_2_ synthesis. In this technique, generally low-melting-point salt has been used as reacting media with other high-melting-point precursors. In a few studies, µm-sized rutile TiO_2_ with a high aspect ratio has been synthesized by the molten-salt method [16,17,18,19]. In those studies, NaCl salt was usually used as a reacting media either alone or together with another salts such as dibasic sodium phosphate (Na_2_HPO_4_). This type of salt combination, known as eutectic composition, is helpful in increasing the reactivity and ion mobility in reacting media at the minimum required temperature [18]. Kim et al. reported eutectic composition of NaCl salt and sodium hexametaphosphate (NaPO_3_)_6_ for the synthesis of 1-D TiO_2_ [20]. It has been explained that the oxide material’s solubility increases by Lux−Flood acid-base interaction; (NaPO_3_)_6_ produces PO_3_- ions which are responsible for a strong Lux−Flood acid that reduces the O^2-^ activity in the system. It creates the reducing atmosphere in the system, which further helps to take O_2_ out of the TiO_2_ crystal. Therefore, anatase TiO_2_ becomes unstable and dissolves in this molten-salt method. Finally, with bond breaking and rearrangements of atoms, it was converted into rutile TiO_2_ rods [20].

However, obtaining uniform size of 1D nano- or micro-particles at a large scale is still highly challenging. Kim et al. in [20] introduced extra additives (i.e., Na_3_P_4_O_7_) with various combinations along with TiO_2_ precursors to obtain a uniform size of rutile TiO_2_ rods.

In this report, the molten-salt synthesis technique was chosen to synthesize rutile-type TiO_2_ microrods with TiO_2_-NaCl media to control size and shape with 1D growth in the air atmosphere. This technique shows many advantages, such as a homogeneous mixture of TiO_2_-NaCl at low temperature, and an easy post-process to remove impurities. Mixtures of two different types of sodium phosphate were used as additives for the crystal growth control. Finally, synthesized rutile-type TiO_2_ microrods were used for detailed study related to size-controlled morphology, crystallinity, and optical properties.

## 2. Experimental Procedures

### 2.1. Materials

Titanium (IV) oxide (rutile seed) was provided by Jiangsu Hehai Nanometer Science & Technology Co., Ltd. (Taixing, Jiangsu province China). Titanium (IV) oxide (anatase powder) (98+%) was purchased from Sigma-Aldrich, Korea. Sodium pyrophosphate decahydrate (97%) and sodium hexametaphosphate (extra pure) were purchased from Alfa-Aesar (Seoul, Korea). Sodium chloride (99.5%) was purchased from Duksan (Ansan-si, Korea). Deionized (DI) water was taken from a Milli-Q IQ 7000 system (ρ >18.2 MΩ.cm). Ethanol (99.5%), acetone (95%), and hexane (95%) were purchased from Samchun (Pyeongtaek, Korea).

### 2.2. Preparation of TiO_2_ Microrods

TiO_2_ microrods were prepared by the molten-salt method [20]. Generally, TiO_2_ rutile seeds and TiO_2_ anatase (ratios vary from 1:1, to 1:2., 1:3, and 1:4) were mixed and ground together with a constant ratio of NaCl and sodium phosphate additives (NaCl: (NaPO_3_)_6_: Na_3_P_4_O_7_ = 4:1:1). To choose the proper ratio of (NaCl: (NaPO_3_)_6_: Na_3_P_4_O_7_), we performed a few experiments with different ratios and checked SEM data. The obtained SEM data are presented in Supporting Information, Figure S1. The actual weight of the precursors in each ratio is presented in the Supporting Information (Table S1). The ratio 4:1:1 showed uniform TiO_2_ particles compared to other ratios. The mixture powder was pelletized using a 13 mm pelletizer (PIKE technologies) at 250 kg.cm^−2^, for 30 s. The pelletized sample was then annealed at 900 °C for 6 h (5 °C/min heating rate) in a box furnace. After that, the pellets were ground to powder and washed with water 3 times using centrifugation at 8000 rpm, 30 min each time, then washed with ethanol and acetone to remove the water. The powder sample was collected, dried at 80 °C for 1 day, and stored at room temperature for further characterization.

### 2.3. Characterization

**Figure 1:**
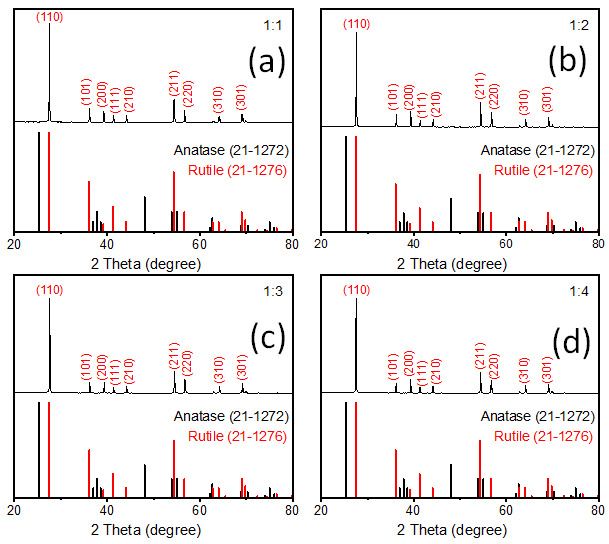
Typical XRD patterns of rutile TiO_2_ microrod samples prepared by different precursor ratios of rutile and anatase TiO_2_ (rutile, seed): TiO_2_ (anatase) (**a**) 1:1, (**b**) 1:2, (**c**) 1:3, and (**d**) 1:4.

The crystal structure of the formed products was characterized by powder X-ray diffraction (Rigaku, MiniFlex 600) using Cu Kα radiation with a wavelength (λ) of 1.5406 Å. The prepared TiO_2_ powder was placed on an XRD sample holder and measured spectra were in the range of 10 to 90 degree of 2 theta with scan rate 2 degree/min. The obtained spectra were plotted in the range of 20 to 80 degrees of 2 theta and are presented in Figure 1. The morphology of the nanostructures was observed by scanning electron microscopy (FE-SEM, JSM-7100F JEOL) equipped with energy dispersive X-ray spectroscopy (EDS). For the SEM measurement, TiO_2_ powder was attached on the SEM holder and to obtain a high-quality picture a thin layer (~10 nm) of Pt was coated via sputter coater. Optical properties were studied using a UV−visible NIR spectrophotometer by Varian Cary with diffuse reflectance mode. In the diffuse reflectance spectroscopy (DRS), a sample holder was filled with prepared TiO_2_ powder and before starting the measurement, baseline correction was done using barium sulfate. After that, each sample was measured in reflectance mode in a wavelength range 250 to 800 nm. An Hitachi F-7000 fluorescence spectrophotometer was utilized to monitor room temperature PL emission. A 355 nm excitation wavelength was used to check the emission spectra in the 400 to 900 nm wavelength range. For the measurement of excitation spectra, 825 nm emission wavelength was used, and excitation was observed in the range of 300 to 380 nm. IR spectroscopy was also observed using the Nicolate Avatar 330 FTIR instrument. For the sample measurements, attenuated total reflectance (ATR) mode was used, and background correction was done without any sample on a diamond crystal with pressure anvil. The sample powder was simply placed on the diamond crystal and pressure was applied through an anvil to start the measurements in the range of 4000 to 600 cm^−1^.

## 3. Result and Discussion

The solution of the TiO_2_ growth process consisted of four chemicals, rutile TiO_2_ seeds, anatase TiO_2_, sodium chloride, and phosphate salts as an additive. The rutile seeds and anatase TiO_2_ precursor particle size were observed as ≤ 50 nm and ≤ 150 nm, respectively, as presented in Figure S2. The rutile seeds were used as nucleation for rod growth because they are stable at high temperature (>800 °C). Sodium chloride acted as the media environment for the TiO_2_ growth process at high temperature (>800 °C). Sodium phosphate ((NaPO_3_)_6_) acted as a Lux−Flood acid, which created a further reducing environment in the system that caused removal of O_2_ from anatase TiO_2_. Hence, anatase TiO_2_ became unstable and dissolved, acting as a titania source for further growth of rutile TiO_2_. The mixture of two sodium phosphate additives ((NaPO_3_)_6_ and Na_3_P_4_O_7_) was useful to create a Lux−Flood acid/base with different Ka (acid dissociation constant) values, helping to increase the solubility and dissolution of anatase TiO_2_ and control the aspect ratio of the TiO_2_ rods [20]. It was expected to control the thickness of the rutile TiO_2_ in the range of micrometers with the combination of these two phosphate salts.

To maintain a homogeneous diffusion, a large quantity of NaCl was used, four times higher than the total TiO_2_ precursor (TiO_2_ (rutile + anatase): NaCl = 1:4). When the ratio of TiO_2_ (rutile) vs TiO_2_(anatase) was kept at 1:1 and the relative ratio of additives varied (ratio of (NaPO_3_)_6_: Na_3_P_4_O_7_ varied from 1:2 to 4:2), it was observed that the size of particles was irregular in all cases. This indicates that the ratio of anatase and rutile was still large, so that the seeds could not grow to consume all the anatase. Instead, the remaining anatase dissolved at high temperature and formed irregular rutile particles. Hence, we further varied the rutile anatase ratio from 1:1, to 1:2, 1:3, and 1:4 by keeping the NaCl: (NaPO_3_)_6_: Na_3_P_4_O_7_ ratio constant as 8:2:2.

**Figure 2:**
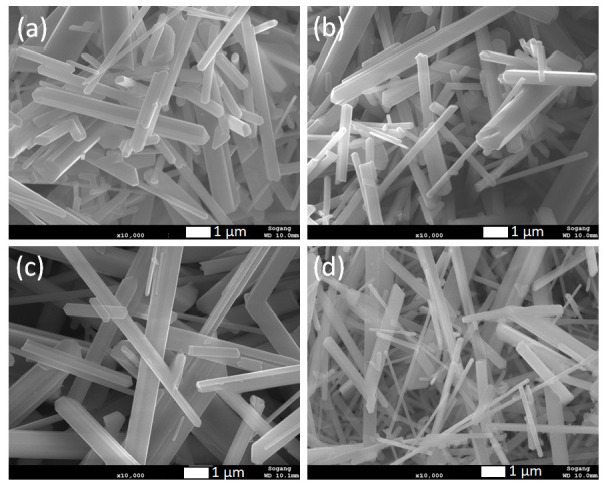
SEM images of rutile TiO_2_ microrod samples prepared by different ratios of TiO_2_ (rutile, seed): TiO_2_ (anatase) as a precursor (**a**) 1:1, (**b**) 1:2, (**c**) 1:3, and (**d**) 1:4.

XRD patterns of synthesized rutile TiO_2_ microrods with different combinations of rutile and anatase precursors are presented in Figure 1. The results were compared with standard JCPDS cards of rutile TiO_2_ (21-1276) and anatase TiO_2_ (21-1272). All the samples showed diffraction peaks at 2 theta values 27.5, 36.1, 39.2, 41.2, 44.05, 54.3, 56.6, 64, and 69° corresponding to crystal planes (110), (101), (200), (111), (210), (211), (220), (310), and (301) of rutile TiO_2_ (21-1276), respectively. There was no single peak observed regarding anatase TiO_2_ (21-1272), so it seems that all the samples were purely crystalline and had a single phase of rutile TiO_2_ with tetragonal crystal structure. In all samples, it was observed that (110) peak intensity was comparatively much higher than the second high intense peak (211). The standard card shows first and second high intense peak ratios, i.e., (110)/(211) is equal to 1.25, and the same peaks area ratio was found in prepared TiO_2_ microrods around 3.58, 3.64, 4.05, and 4.35 for sample 1, 2, 3, and 4, respectively, which were significantly higher than the rutile JCPDS card data. This indicates that TiO_2_ particles grow in one axis direction with high surface area along [110], which is also known as the preferred axis (or crystal facet) orientation growth. These results are quite consistent with SEM data (Figure 2), which shows one-axis-oriented TiO_2_ microrods in all four prepared samples. However, Figure 2a and Figure S3a in Supporting Information show samples prepared at (1:1) ratio of rutile and anatase TiO_2_ precursors appeared with nanorods having different sizes of ~550 to 700 nm in diameter and from ~30 to 50 µm in length. All particles appeared with a smooth surface without further growth or deposition. When the amount of anatase increased and the ratio was (1:2), microrods appeared with smooth and clean surfaces (Figure 2b and Figure S3b). The shape and size of these rods was not much different than that of the samples with a (1:1) ratio. The TiO_2_ prepared with further increasing anatase precursors at a (1:3) ratio showed relatively uniform microrods (Figure 2c and Figure S3c). The size of rods increased to nearly 550–700 nm in diameter with a length from 30 to 50 µm. When rutile and anatase ratios increased to 1:4, non-uniform microrods were shown with random sizes and lengths; approximately 200 nm–2 µm diameter range and 10–50 µm length range (Figure 2d and Figure S3d). The rutile TiO_2_ prepared with a ratio of 1:3 showed microrods comparatively uniform in shape and size. To identify other impurities, SEM-EDS measurements were carried out for all synthesized rutile TiO_2_ samples, and are presented in Supporting Information Figure S4. It was observed that no other impurities were present in prepared samples.

**Figure 3:**
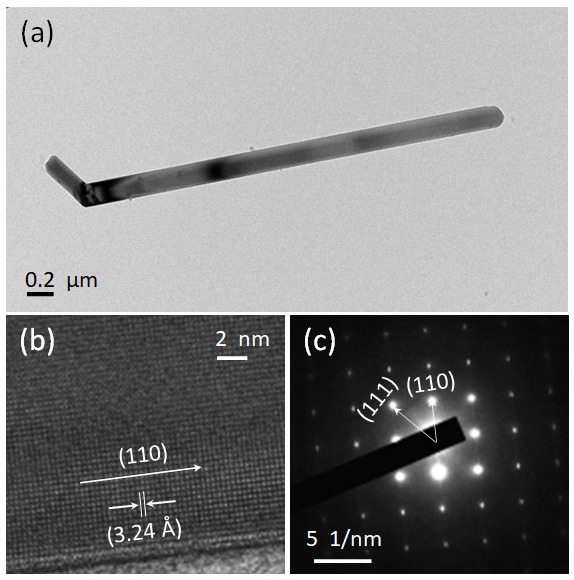
(**a**) TEM, (**b**) HRTEM and (**c**) SAED images of rutile TiO_2_ microrod samples prepared with a 1:3 ratio of TiO_2_ (rutile, seed): TiO_2_ (anatase) as a precursor.

Figure 3 shows the TEM, HRTEM, and SAED patterns of synthesized rutile TiO_2_ microrods prepared with rutile and an anatase ratio 1:3. The TEM image shows a clear picture of synthesized microrods with diameter of more than 0.2 µm and length more than 2 µm. Figure 3b shows a selective area electron diffraction (SAED) pattern, and it shows clear lattice points, which are signs of single crystallinity with growth direction along [110] and [111]. In further observation, a high-resolution TEM (HRTEM) image presented in Figure 3c shows clear lattice fringes with an observed interplanar distance of 3.24 Å along the (110) plane. These data are additional confirmation of preferred orientation and growth along the [110] direction, the same as XRD.

**Figure 4:**
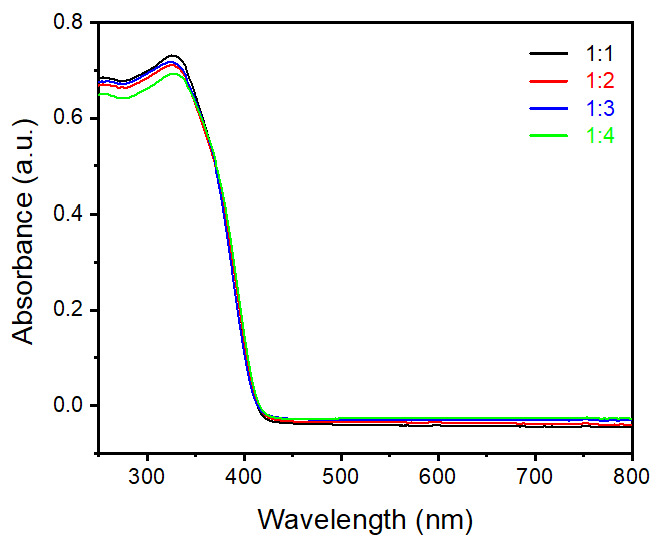
UV−visible absorbance spectra of rutile TiO_2_ microrod samples prepared by different ratios of TiO_2_ (rutile, seed): TiO_2_ (anatase) as a precursor (black) 1:1, (red) 1:2, (blue) 1:3, and (green) 1:4.

**Figure 5:**
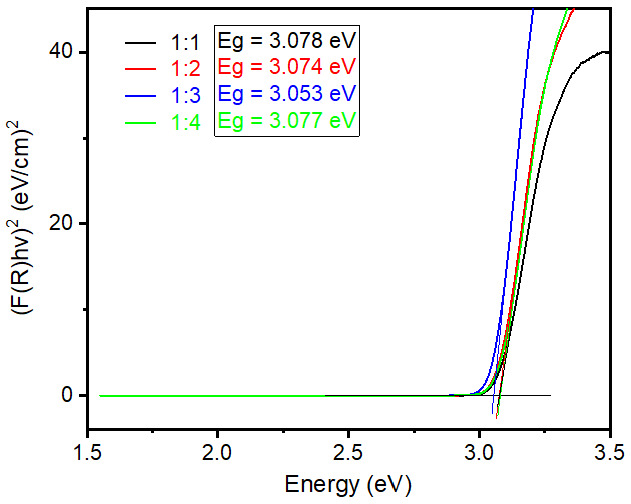
Band gap energy calculation using Tauc plot method for rutile TiO_2_ microrod samples prepared by different ratios of TiO_2_ (rutile, seed): TiO_2_ (anatase) as a precursor (black) 1:1, (red) 1:2, (blue) 1:3, and (green) 1:4.

Figure 4 shows UV−visible absorption spectra of samples prepared by different ratios of rutile and anatase TiO_2_ as precursors. Typical TiO_2_ materials show an absorbance range until 400 nm wavelength; the same phenomenon was observed in all four synthesized samples and is presented in Figure 4. In general, nanoparticle sizes less than 100 nm show a slight variation in absorption range due to changes in band gap energy [21]. However, in this case particle sizes are quite high in the µm range, hence we did not observe any changes in light absorption. Typically, the value of band gap energy was calculated by using Tauc and Davis−Mott relation, with the equation given below (Equation (1)), [22,23].

(1) $$h=K{\left(h-Eg\right)}^{n}$$

where *α* = absorption coefficient, *h* = Plank’s constant, ν = frequency, *K* = energy independent constant, *Eg* = band gap energy, “*n*” is a nature of transmission and depends upon the materials selection rules regarding electron transition; e.g., 1/2 for allowed direct transition, 3/2 for forbidden direct transition, 2 for allowed indirect transition, and 3 for forbidden indirect transition. Rutile TiO_2_ shows direct electronic transition, hence *n* = ½ was considered in this case. However, with unknown thickness due to TiO_2_ as powder form, finding “*α*” value was difficult with UV absorption spectra. To avoid this problem, diffuse reflectance spectroscopy (DRS) was used for further study. The theory of DRS is based on the Kubelka−Munk equation (Equation (2)): [24,25,26,27].

(2) $$\frac{\alpha }{s}=\frac{{\left(1-R\right)}^{2}}{2R}=F\left(R\right)$$

where “*α*” stands for absorption coefficient, “*s*” for scattering coefficient and these two values changes with shape, size, and packing of materials. The reflectance of the materials is denoted as *R*. In practice, the measured diffuse reflectance spectrum is the ratio of the analyzed sample’s reflection intensity to the standard sample’s reflection intensity. In the Kubelka−Munk function, *F(R)* is the conversion of reflectance data, which equals to the absorption coefficient (*α*) per unit scattering (*s*). Because scattering is assumed to be relatively constant for all the wavelengths, the absorption coefficient (*α*) is directly proportional to the *F(R)* value. Finally, to identify the band gap energy value of synthesized rutile TiO_2_ microrods, diffuse reflectance spectra were collected for all the samples and plotted in a graph [F(R)hv]^2^ vs. hv, presented in Figure 5. Tauc plot extrapolation determines the value of band gap energy as around 3.06 eV (±0.01 eV) for all synthesized rutile TiO_2_ microrods. The separate graphs of Tauc plot extrapolation are presented in Supporting Information as Figure S5.

**Figure 6:**
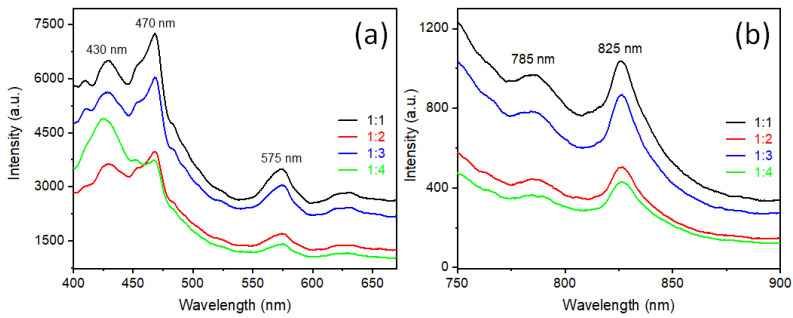
Photoluminescence emission spectra of synthesized rutile TiO_2_ prepared with different precursors ratio of rutile: anatase TiO_2_ : (black) 1:1, (red) 1:2, (blue) 1:3, and (green) 1:4. (**a**) PL emission range from 400 to 675 nm and (**b**) PL emission range from 750 nm to 900 nm at excitation wavelength 355 nm.

Room temperature photoluminescence (PL) measurements were done with an excitation wavelength 355 nm of a Xenon lamp. The emission spectra of all synthesized rutile TiO_2_ nanorods were observed in the range between 400 to 900 nm. The excitation spectra are presented in Supporting Information as Figure S6. In the complete spectra (Figure S7), a secondary harmonic high intense peak is presented at 710 nm, which is exactly twice the excitation wavelength (355 nm). For clearer visualization, these spectra are presented in two different regions: a range from 400 nm to 675 nm (Figure 6a) and a range from 750 nm to 900 nm (Figure 6b). The peak at 430 nm is observed due to recombination of free electrons and holes near the band edge of rutile TiO_2_ [28]. In the middle of visible range, two emission peaks at 470 nm and 575 nm were observed due to radiative transition of the self-trap electron-hole recombination. The self-trap state is due to the oxygen vacancy present in TiO_2_. When a sample anneals at high temperature, oxygen vacancies are created inside the materials [29]. As describing in the experimental section, the sample was pelletized and annealed at 900 °C; therefore, there is a high chance of creating oxygen vacancy inside the rutile TiO_2_ particles. The PL emission spectra in Figure 6a are presented in the visible range and near the UV region, which is quite similar to the anatase phase [30], but there is a possibility that rutile TiO_2_ can show emission spectra similar to anatase due to oxygen vacancy [31]. Figure 6b shows typical emission spectra of rutile TiO_2_, which shows PL emission at 825 nm and a small hump at 785 nm. The rutile TiO_2_ shows an emission range near the IR (NIR) region, due to 1) radiative recombination of trapped hole with free electrons or 2) radiatively recombination of trapped electrons with a free hole [30]. In the rutile TiO_2_ crystal structure, along the (110) and (100) plane are shown threefold coordinated oxygen atoms. When light is incident on rutile TiO_2_, electrons are excited to conduction band level and holes are created in the valence band, those generated holes transferred towards the surface, but due to threefold coordinated oxygen atoms few of those holes are trapped near to the (110) or (100) plane/surface [32,33,34]. That trapped energy level, known as a self-trapped hole (STH), is quite low in energy state and matched with the near-IR range [30]. As we have observed in XRD, SEM, and TEM samples, synthesized rutile type TiO_2_ microrod samples in this report show preferred orientation along the [110] direction and rod-type morphology. Hence, they have a larger site of self-trapped holes, which are responsible for PL emission at 825 nm and 785 nm wavelengths.

**Figure 7:**
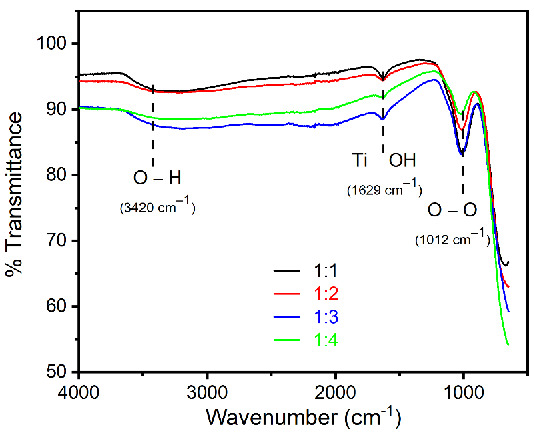
FTIR spectra for rutile TiO_2_ microrod samples prepared by different ratios of TiO_2_ (rutile, seed): TiO_2_ (anatase) as a precursor (black) 1:1, (red) 1:2, (blue) 1:3, and (green) 1:4.

In the synthesis experiments, sodium phosphate was used as an additive, therefore it could be present as impurities in the final product. Hence, FTIR spectra were measured to identify any type of impurities of synthesized rutile TiO_2_ microrods with different ratios of titania precursors. The observed data (Figure 7) show typical spectrum of rutile TiO_2_ microrods with the hydroxyl group (OH) on the surface. It is well known that air moisture (hydroxyl group) can be easily attached on the surface of rutile TiO_2_ microrods, and it is called a surface hydroxyl group [35]. Hence, the broad peaks at around 3420 cm^−1^ show stretching vibration of the OH group. A peak position at around 1629 cm^−1^ represents OH bending vibration on the rutile TiO_2_ microrod surface [36]. The peak appearing at 1012 cm^−1^ is a characteristic peak of O-O stretching vibration [36]. However, no impurity peaks regarding phosphate were observed in all synthesized TiO_2_ microrods.

## 4. Conclusions

- It is concluded that micrometer-length TiO_2_ rods were successfully synthesized by using the molten-salt method using TiO_2_(rutile: anatase)/NaCl/Na(PO_3_)_6_/Na_3_P_4_O_7_ as precursors.
- In the molten-salt precursors, rutile TiO_2_ acted as nuclei for rod formations and anatase TiO_2_ acted as a source of titanium (Ti) for rutile rod growth in the presence of NaCl as reacting media with eutectic composition of sodium phosphates (Na(PO_3_)_6_/Na_3_P_4_O_7_).
- For proper eutectic composition, five different ratios of NaCl/Na(PO_3_)_6_/Na_3_P_4_O_7_ were used and among them only 4:1:1 showed TiO_2_ rods significantly controlled in size and length.
- By keeping the constant ratio of sodium chloride and sodium phosphate (NaCl: Na(PO_3_)_6_:Na_3_P_4_O_7_) as 4:1:1, variation of TiO_2_ precursors (rutile:anatase) was studied and it was found that a (1:3) ratio produced comparatively uniform size and length of TiO_2_ rods.
- The synthesized rutile TiO_2_ showed various emission wavelengths, such as 430, 470, 575, 785, and 825 nm at 355 nm excitation wavelength. Photoluminescence emission was observed due to oxygen vacancy generated at high temperature annealing (900 °C).

## Data Availability

Not applicable.

## Supplementary Materials

The following supporting information can be downloaded at: https://www.mdpi.com/article/10.3390/nano12152626/s1. Figure S1. Synthesized rutile TiO_2_ micro-rods by changing additives ratio ((NaPO_3_)_6_:Na_3_P_4_O_7_) to (a) 4:0, (b) 3:1, (c) 2:2, (d) 1:3 and (e) 0:4 and keeping other precursors ratio constant (rutile : anatase : NaCl = 0.5:1.5:8); Figure S2. Low and high magnification SEM images of TiO_2_ precursors (a,a’) anatase TiO_2_ and (b,b’) rutile TiO_2_; Figure S3. SEM images with lower magnification of rutile TiO_2_ micro-rods samples prepared by different ratios of TiO_2_ (rutile, seed):TiO_2_ (anatase) as a precursor (a) 1:1, (b) 1:2, (c) 1:3 and (d) 1:4; Figure S4. SEM and EDS data of synthesized rutile TiO_2_ rods with different ratios of TiO_2_ (rutile, seed):TiO_2_ (anatase) as a precursor (a,a’) 1:1, (b,b’) 1:2, (c,c’) 1:3 and (d,d’) 1:4; Figure S5. Band gap energy calculation by using Tauc plot method for TiO_2_ sample prepared by different ratios of TiO_2_ (rutile, seed):TiO_2_ (anatase) as a precursor (a) 1:1, (b) 1:2, (c) 1:3 and (d) 1:4; Figure S6. Photoluminescence excitation spectra of synthesized rutile TiO_2_ micro-rods with different combination of rutile:anatase precursors such as (black) 1:1, (red) 1:2, (blue) 1:3 and (green) 1:4. for the emission wavelength 825 nm. Figure S7. Complete photoluminescence spectra of synthesized rutile TiO_2_ micro-rods with different combination of rutile : anatase precursors such as (black) 1:1, (red) 1:2, (blue) 1:3 and (green) 1:4. Secondary harmonic generation peak observed at 710 nm which started from 675 nm and end up at 750 nm. This secondary harmonic peak is exactly twice of excitation wavelength 355 nm. Table S1. Actual weight and related ratios of precursors used for rutile TiO2 synthesis by molten salt method.
