# Supplementary material for: Synthesis of Uniform Size Rutile TiO2 Microrods by Simple Molten-Salt Method and Its Photoluminescence Activity
Source: Nanomaterials (Basel). 2022 Jul 29;12(15):2626. doi: 10.3390/nano12152626 (PMC9370513; doi:10.3390/nano12152626)
Supplement: Supplementary file 1 [file nanomaterials-12-02626-s001.zip › nanomaterials-1781883-supplementary.pdf]

Supporting Information

Synthesis of Uniform Size Rutile TiO_2 Micro-rods by Simple Molten-salt Method and Its Photoluminescence Activity

Hieu Minh Ngo[1$], Amol Uttam Pawar[3$], Jun Tang[2], Zhongbiao Zhuo[2], Don Keun Lee[3], Kang Min Ok[1], and Young Soo Kang[3*]

[1]Department of Chemistry, Sogang University, Seoul, Republic of Korea.
[2]Coloray Technology Development Co., LTD, Deqing City, Zhejiang Province, China.
[3]Environmental and Climate Technology, Korea Institute of Energy Technology, Naju-Si, Jeollanam-do 58219, Republic of Korea
[$] Authors have an equal contribution
* Correspondence: author: Young Soo Kang; E-mail: yskang@kentech.ac.kr

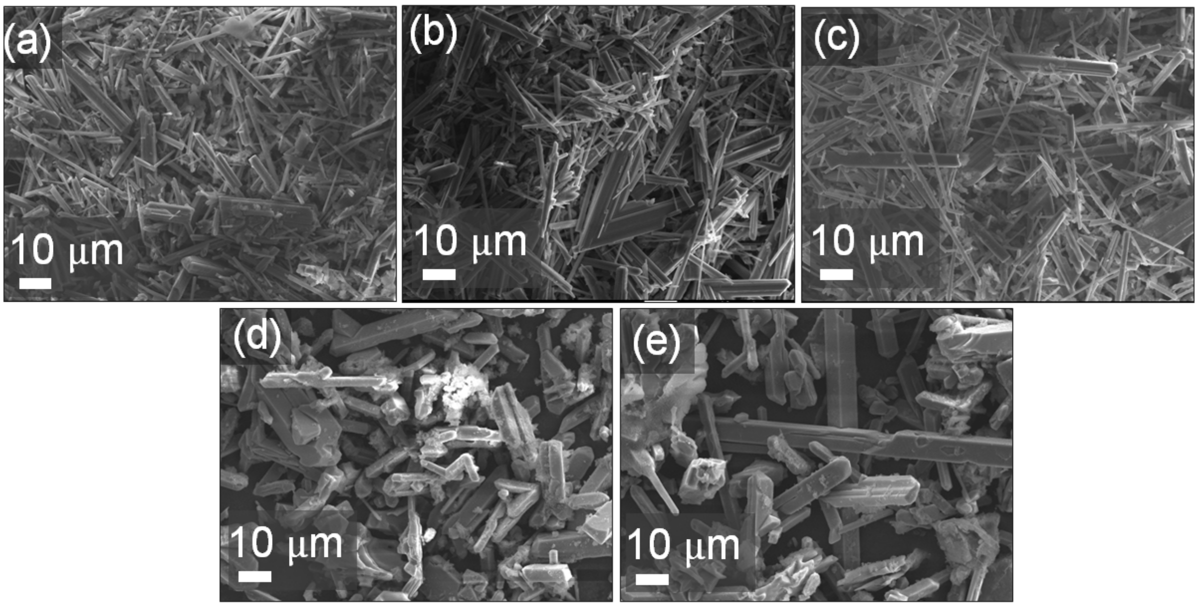

Figure S1. Synthesized rutile TiO_2 micro-rods by changing additives ratio ($(NaPO_3)_6$: $Na_3P_4O_7$) to (a) 4:0, (b) 3:1, (c) 2:2, (d) 1:3 and (e) 0:4 and keeping other precursors ratio constant (rutile : anatase : NaCl = 0.5 : 1.5 : 8).

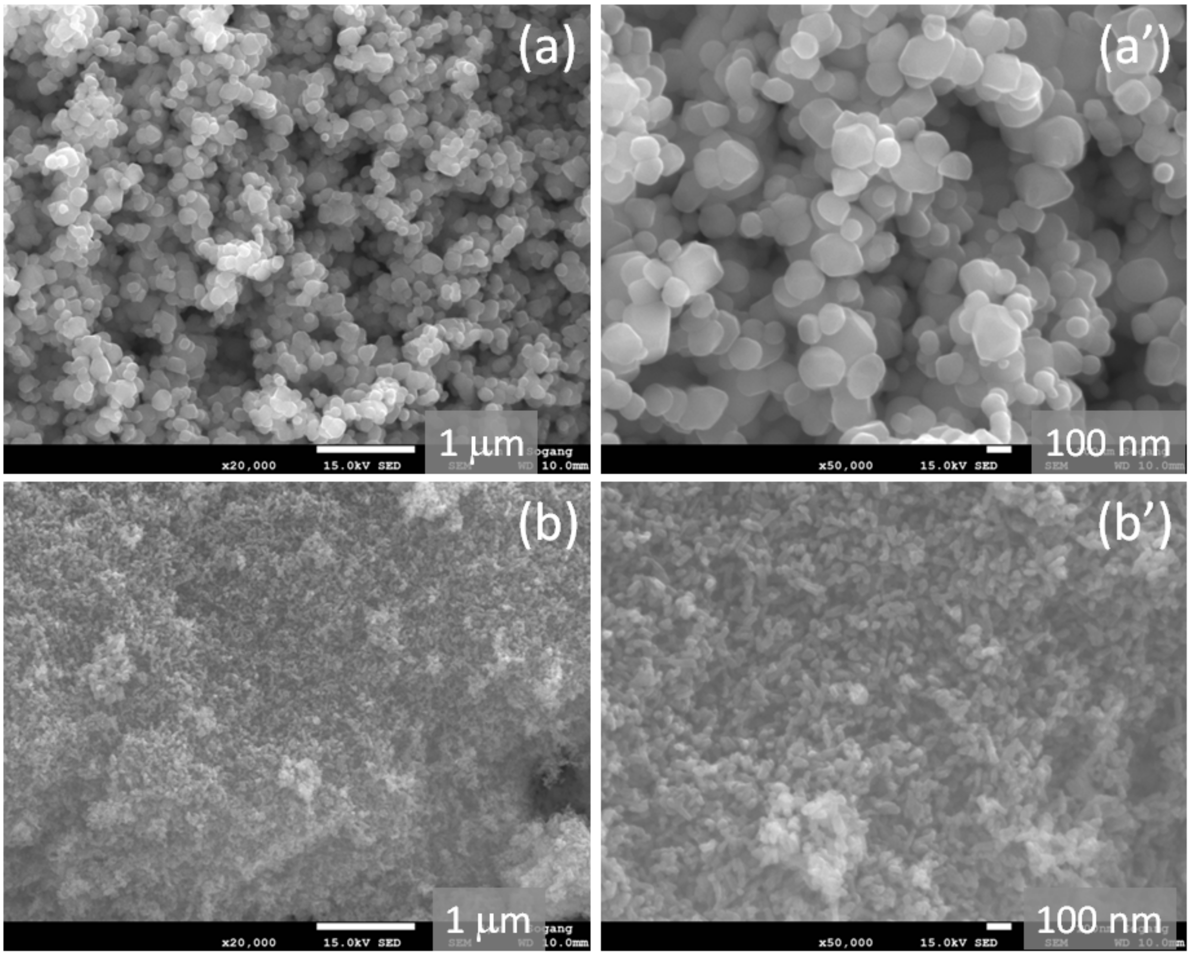

Figure S2. Low and high magnification SEM images of TiO_2 precursors (a, a') anatase TiO_2 and (b,b') rutile TiO_2.

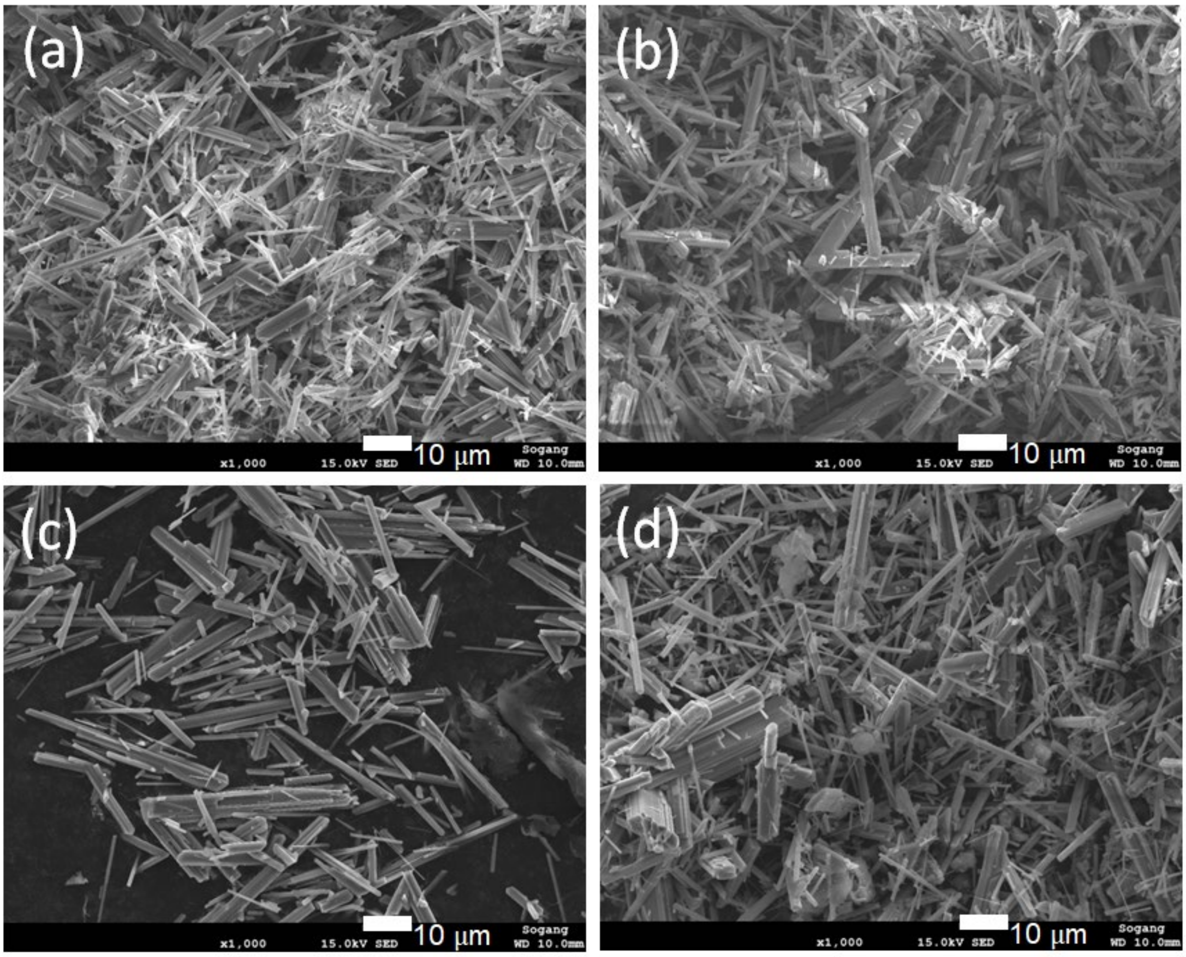

Figure S3. SEM images with lower magnification of rutile TiO_2 micro-rods samples prepared by different ratios of TiO_2(rutile, seed): TiO_2 (anatase) as a precursor (a) 1:1, (b) 1:2, (c) 1:3 and (d) 1:4.

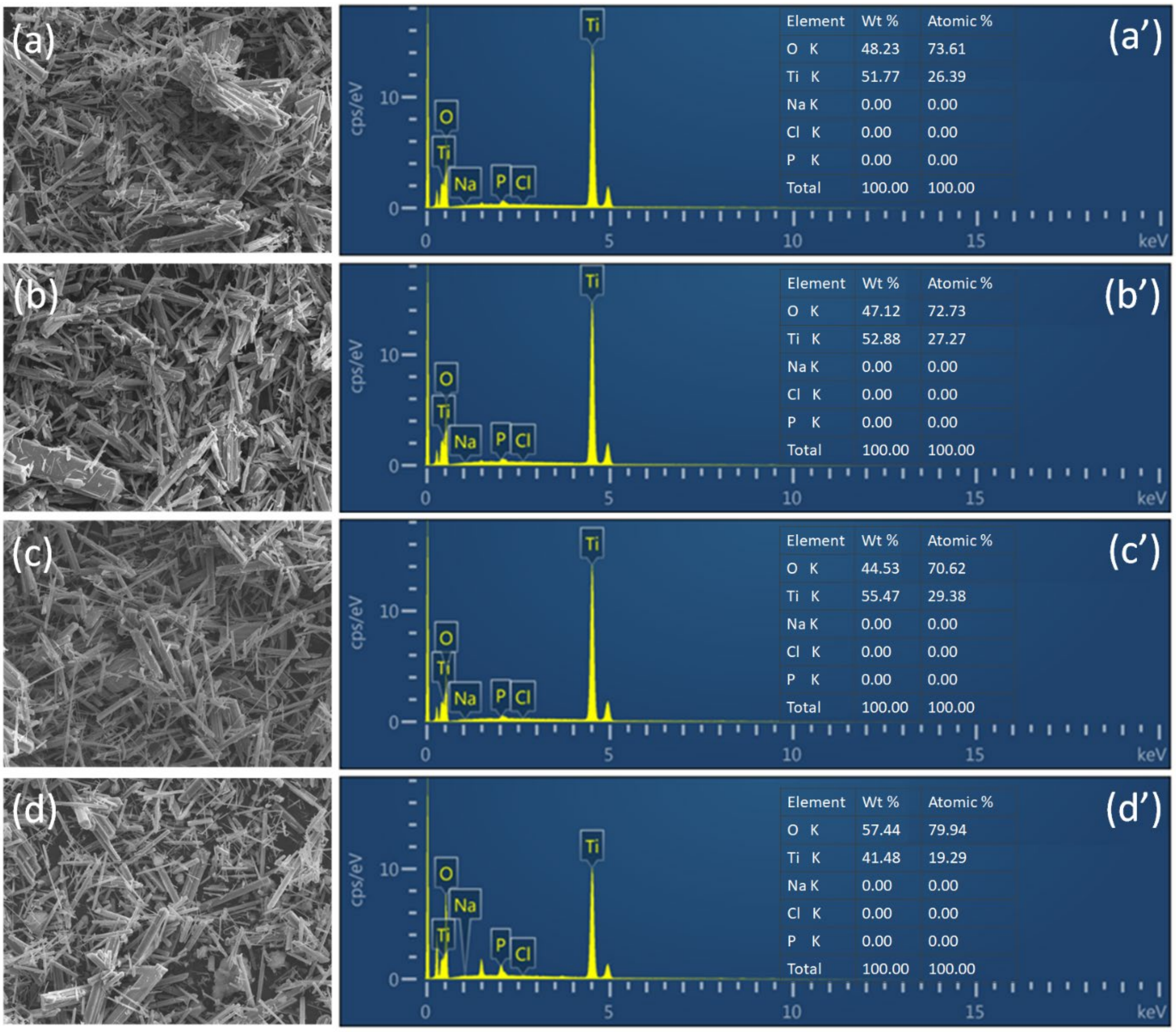

Figure S4. SEM and EDS data of synthesized rutile TiO_2 rods with different ratios of TiO_2 (rutile, seed) : TiO_2 (anatase) as a precursor (a,a’) 1:1, (b,b’) 1:2, (c,c’) 1:3 and (d,d’) 1:4.

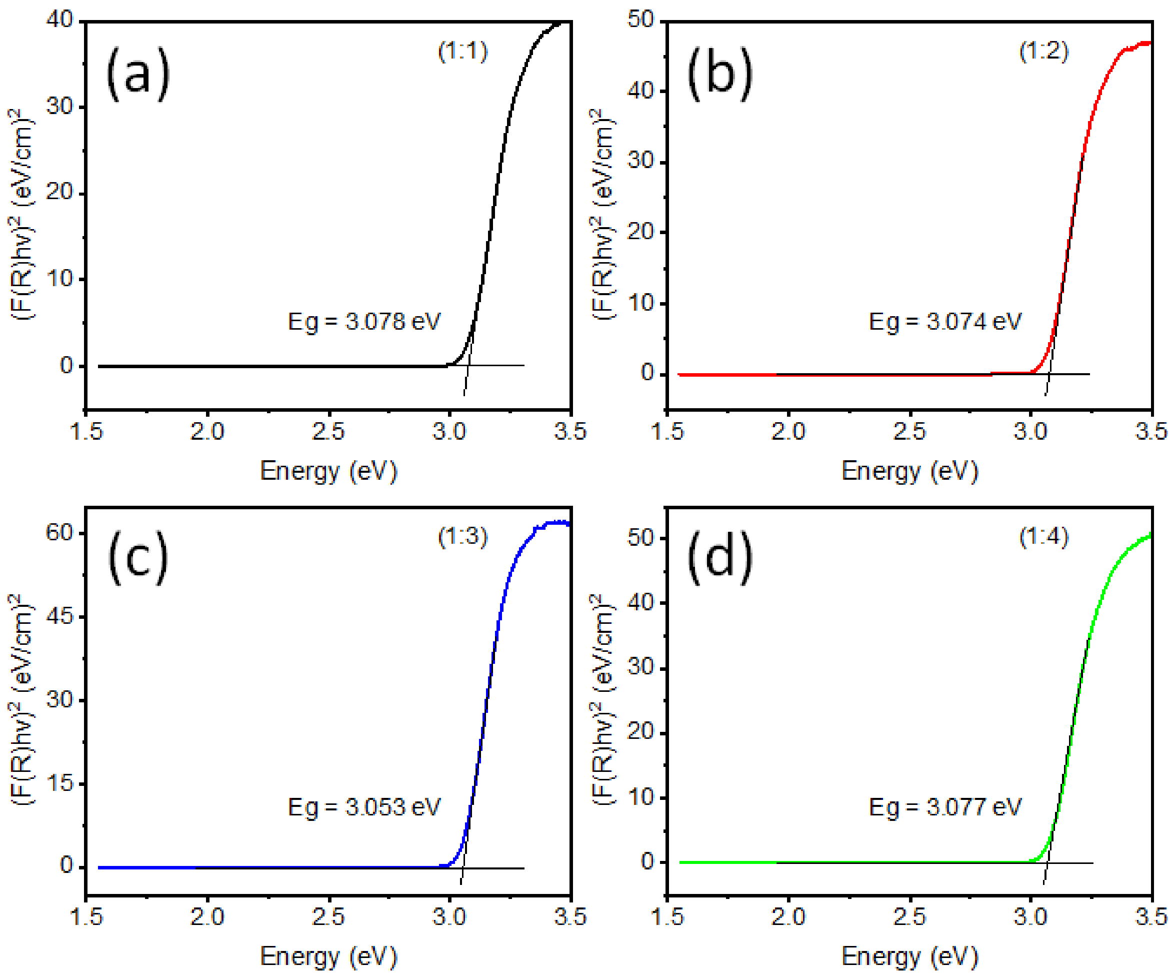

Figure S5. Band gap energy calculation by using Tauc plot method for TiO_2 sample prepared by different ratios of TiO_2(rutile, seed): TiO_2 (anatase) as a precursor (a) 1:1, (b) 1:2, (c) 1:3 and (d) 1:4.

Table S1. Actual weight and related ratios of precursors used for rutile TiO_2 synthesis by molten salt method.

**No**	**Sample ratio**	**Rutile (g)**	**Anatase (g)**	**Ratio (Rutile:Anatase)**	**NaCl (g)**	**$(NaPO_3)_6$ (g)**	**$Na_3P_4O_7$ (g)**	**Additive ratios (NaCl: $Na(PO_3)_6$: $Na_3P_4O_7$)**
**1**	0.4:1.6:8:2:2	0.4	1.6	1:4	8	2	2	4:1:1
**2**	0.5:1.5:8:2:2	0.5	1.5	1:3	8	2	2	4:1:1
**3**	0.67:1.34:8:2:2	0.67	1.34	1:2	8	2	2	4:1:1
**4**	1:1:8:2:2	1	1	1:1	8	2	2	4:1:1
**5**	0.5:1.5:8:2:2	0.5	1.5	1:3	8	4	0	8:4:0
**6**	0.5:1.5:8:2:2	0.5	1.5	1:3	8	3	1	8:3:1
**7**	0.5:1.5:8:2:2	0.5	1.5	1:3	8	2	2	8:2:2 = 4:1:1
**8**	0.5:1.5:8:2:2	0.5	1.5	1:3	8	1	3	8:1:3
**9**	0.5:1.5:8:2:2	0.5	1.5	1:3	8	0	4	8:0:4

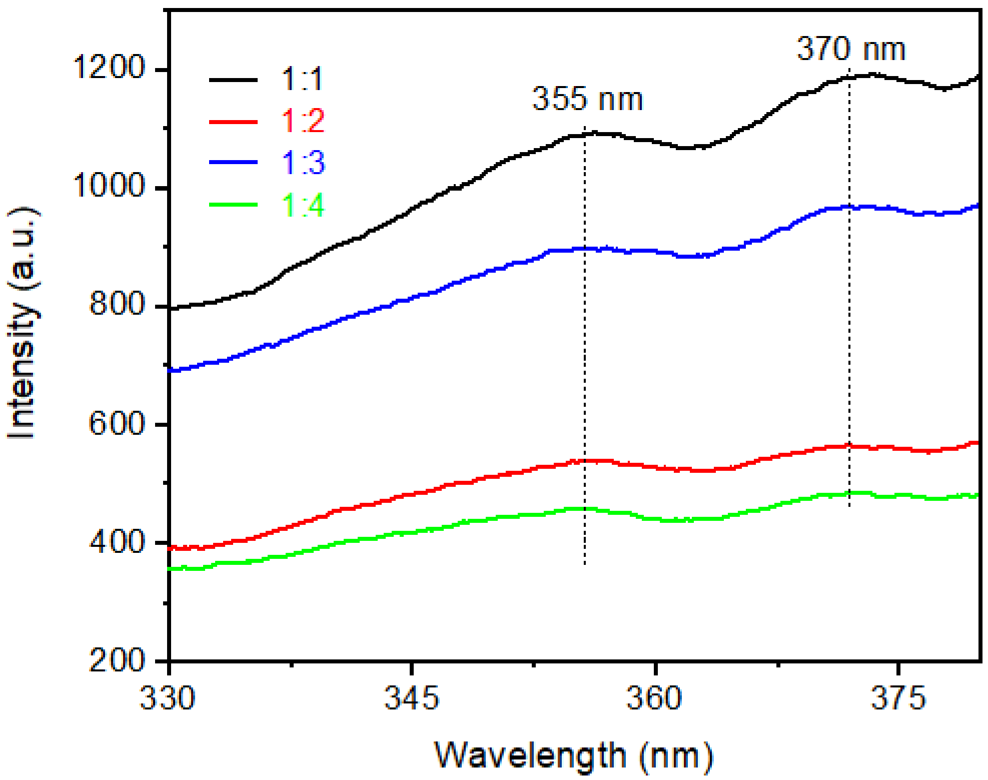

Figure S6. Photoluminescence excitation spectra of synthesized rutile TiO_2 micro-rods with different combination of rutile : anatase precursors such as (black) 1:1, (red) 1:2, (blue) 1:3 and (green) 1:4. for the emission wavelength 825 nm.

Above PL excitation spectra shows two peaks at 355 nm and 370 nm wavelength, however when we measured emission spectrum both those excitation wavelengths show same emission spectrum. Moreover, 355 nm wavelength show little higher intensity peaks compare to 370 nm. Hence, 355 nm wavelength was used as excitation wavelength.

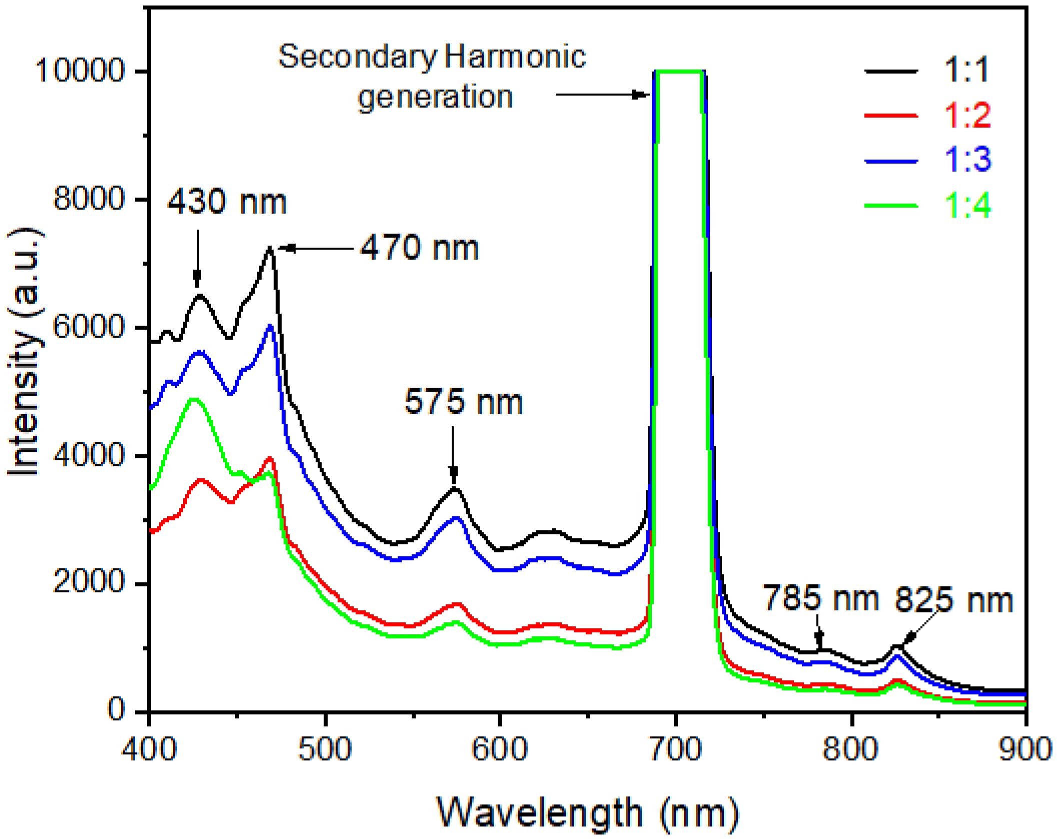

Figure S7. Complete photoluminescence spectra of synthesized rutile TiO_2 micro-rods with different combination of rutile : anatase precursors such as (black) 1:1, (red) 1:2, (blue) 1:3 and (green) 1:4. Secondary harmonic generation peak observed at 710 nm which started from 675 nm and end up at 750 nm. This secondary harmonic peak is exactly twice of excitation wavelength 355 nm.
